# Comparison and Trends of Endovascular, Surgical and Hybrid Revascularizations and the Influence of Comorbidity in 1 Million Hospitalizations Due to Peripheral Artery Disease in Germany Between 2009 and 2018

**DOI:** 10.1007/s00270-022-03136-9

**Published:** 2022-04-15

**Authors:** Josua A. Decker, Magnus Helmer, Stefanie Bette, Florian Schwarz, Thomas J. Kroencke, Christian Scheurig-Muenkler

**Affiliations:** Department of Diagnostic and Interventional Radiology, University Hospital Augsburg, Stenglinstr. 2, 86156 Augsburg, Germany

**Keywords:** Peripheral artery disease, Endovascular revascularization, Surgical revascularization, Hybrid revascularization, Comorbidity

## Abstract

**Objective:**

To analyze trends and differences of endovascular, surgical and hybrid revascularization approaches and the impact of comorbidity on characteristics, costs, and outcome of in-patients with peripheral artery disease (PAD) of the lower extremity.

**Methods:**

Analyzing data provided by the Research Data Center of the German Federal Statistical Office, we included all hospitalizations due to PAD Fontaine IIb (Rutherford 2–3) or higher in Germany between 2009–2011 and 2016–2018. According to the individually performed procedures encoded by the Operation and Procedure Classification System, we divided hospitalizations by revascularization procedures into sole endovascular, sole surgical, hybrid, two-step and no revascularization. Patient’s comorbidity was assessed using the linear van Walraven comorbidity score (vWs).

**Results:**

1,067,671 hospitalizations (mean age 71.3 ± 11.1 years; 60.1% male) were analyzed. Between 2009–2011 and 2016–2018, reimbursement costs rose by 28.0% from €2.72 billion (€5,350/case) to €3.49 billion (€6,238/case). The share of hospitalizations with any revascularization increased by 8.9% (67.7–73.7%) driven by an increase in two-step (+ 63.3%), hybrid (+ 58.2%) and sole endovascular revascularizations (+ 32.6%), while sole surgical approaches declined (− 18.2%). Hospitalizations of more comorbid patients (vWs ≥ 20) rose by 46.8% (21,444–31,478 cases), showed an overproportionate increase in costs of 124.6% (+ €1,750/case) and were associated with more individual procedures (+ 90.6%).

**Conclusions:**

In-patient treatment of PAD patients shows increasing numbers of hybrid and sole endovascular revascularizations and more patients with higher comorbidity, while sole surgical interventions and in-hospital mortality decrease. Consequently, associated costs are surging especially in more comorbid patients due to an increasing number of performed procedures and escalation of therapy.

**Supplementary Information:**

The online version contains supplementary material available at 10.1007/s00270-022-03136-9.

## Introduction

Affecting over 200 million people worldwide, peripheral artery disease (PAD) ranks third in atherosclerotic causes of morbidity following coronary artery disease and cerebrovascular disease [1, 2]. Being already substantial, the socioeconomic burden of PAD continues to increase due to the aging of society and the rise of promoting risk factors such as type 2 diabetes mellitus or dyslipidemia [3–5].

Although medication, smoking cessation and exercise significantly improve the prognosis of PAD in earlier stages of the disease, interventional revascularization procedures are performed in a high percentage of patients with more advanced PAD stages [6–8]. Supported by technical advantages, favorable outcomes, and lower procedural costs, the endovascular treatment of PAD overcame sole surgical treatment and emerged to the dominant revascularization approach over the past decades [9–13].

Although several studies reported a cost benefit of endovascular revascularizations compared to surgical approaches, the healthcare costs for the treatment of PAD are still high and continue to rise, especially in patients with higher comorbidity [4, 5, 9, 14, 15]. Additionally, hybrid revascularization procedures have also recently been shown to increase in number and provide perioperative advantages to open surgical revascularization [16–18]. Overall, robust large-scale data to understand how the surge in resulting costs can be explained and what the role of different revascularization approaches (such as hybrid interventions) is, are lacking.

In this study, we therefore analyzed procedural trends and compared different revascularization approaches in the in-patient setting of PAD patients with special focus on resulting costs and primary outcome over a whole decade. Furthermore, we aimed to identify reasons that led to the observed increase in costs and changes of accompanying outcomes, especially in patients with multiple comorbidities.

## Materials and Methods

### Data Source

Data were obtained from the Research Data Center (RDC) of the German Federal Statistical Office (Destatis) [19]. We analyzed characteristics, procedures and in-hospital outcomes of patients admitted due to PAD in the years 2009–2011 and 2016–2018. All in-patient treatments in German hospitals excluding psychosomatic and psychiatric hospitals are summarized in this dataset. Using dedicated syntaxes written by the authors, analyses were remotely conducted by Destatis and the results were transferred after they passed an anonymity check that censored subgroups with fewer than five individual cases. Three-year periods were compared to avoid censoring of subgroups with lower number of hospitalizations and to obtain a comprehensive overview.

### German Diagnosis Related Groups (G-DRG) Remuneration System

In order for the hospitals to receive adequate compensation, the remuneration institute InEK (Institut für das Entgeltsystem im Krankenhaus) assigns each hospitalization to a case-specific DRG determined by a combination of main and secondary diagnoses, and the performed procedures. The main diagnosis (reason for admission) and any secondary diagnoses (comorbidities) are encoded using the International Classification of Diseases 10th Revision in its German modification (ICD-10-GM). The procedures performed during the hospitalization are coded using the Operation and Procedure Classification System (OPS). All the encoded case-specific ICD-10 codes, OPS codes and corresponding DRG were available in the analyzed dataset.

### Patient Cohort and Subgroup Analysis

All hospitalizations due to PAD Fontaine stage IIb (pain-free walking distance < 200 m; corresponds to Rutherford category 2–3) or higher stages in the years 2009–2011 and 2016–2018 were included. Using individual OPS codes, each hospitalization was divided into the following categories: (1) No revascularization; (2) endovascular revascularization; (3) open surgical revascularization; (4) two-step revascularization; (5) hybrid revascularization. Hybrid approaches were defined by an individual hybrid-code that defines a joint surgical and endovascular procedure in one intervention. Two-step approaches were assigned when surgical and endovascular were performed in one hospitalization but not in one single intervention. The accompanying comorbidity burden was assessed by calculating the linear van Walraven score using ICD-10 codes [20, 21]. Detailed listing of the analyzed ICD-10 codes and OPS codes used for selection and subgroup analysis is provided in the Supplemental material. The most relevant DRGs that accounted for 80% of all hospitalizations were determined. Overall and subgroup-specific case mix indices were calculated using the respective cost-weight factor, simplified by using the factor which was allocated in 2011 for the time period 2009–2011 and in 2018 for the period 2016–2018, respectively. Every year, a base rate is determined for DRG calculation. Depending on the severity and complexity of a case, this is assigned to a specific DRG. This DRG is assigned a certain cost-weight factor that determines how much of the base rate can be charged as remuneration. The sum of all cost-weight factors is the case mix. The case mix index describes the average severity of patient cases, measured on a scale corresponding to the global expenditure of all hospital cases. It is calculated by dividing the case mix by the number of patients.

### Statistics and Data Analysis

Data analysis and coding for controlled remote data processing were performed using R version 4.1.0 (https://www.r-project.org/). Calculation of the weighted linear van Walraven score was performed using the R package *comorbidity* (https://cran.r-project.org/package=comorbidity) [22]. Categorial variables are presented as absolute numbers (*n*) and percentages (%); continuous variables are presented as mean or median with standard deviation (SD) or interquartile range (IQR) as indicated.

## Results

**Fig. 1:**
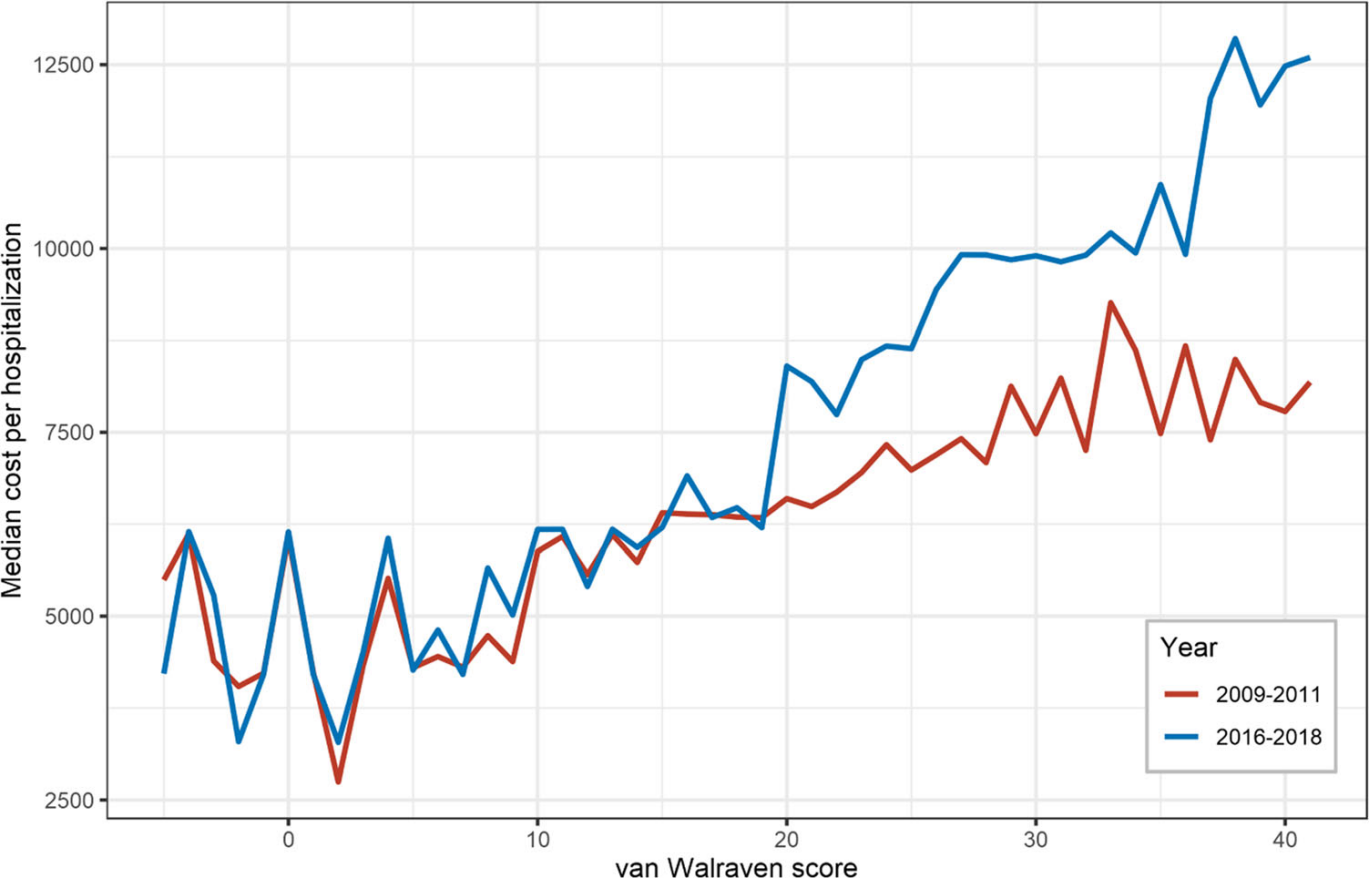
Correlation between median costs (in €) per hospitalization and the linear van Walraven comorbidity score of patients hospitalized due to peripheral artery disease between the time periods 2009–2011 and 2016–2018

A total of 1,067,671 hospitalizations (mean age 71.3 ± 11.1 years; 60.1% male) were analyzed in this study. Between the time periods 2009–2011 and 2016–2018, the number of hospitalizations increased by 9.8% from 508,886 to 558,785. Within the same timeframe, the associated reimbursement costs rose by 28.0% from €2.72 billion (€5,350 per case) to €3.49 billion (€6,238 per case) with a notable increase in the median cost per hospitalization with a van Walraven comorbidity score ≥ 20 (Fig. 1). Mean in-hospital stay decreased by 16.1% from 11.5 ± 10.9 to 9.6 ± 9.3 days, and in-hospital mortality decreased from 3.0 to 2.6% from 2009–2011 to 2016–2018, respectively. Detailed patient characteristics and their development between 2009–2011 and 2016–2018 are shown in Table 1. Primary in-hospital outcome data are presented in Table 2.

**Table 1:** Characteristics of patients admitted due to PAD and changes from 2009–2011 to 2016–2018

Characteristic	Font	Treatment	2009–2011	2016–2018	Abs. change	Relative%
Hospitalizations	IIb	ev	129,697 (53.6)	163,668 (62.4)	+ 33,971 (+ 26.2%)	+ 16.5
		Surgical	49,517 (20.4)	41,055 (15.7)	− 8462 (− 17.1%)	− 23.4
		Two-step	6223 (2.6)	11,225 (4.3)	+ 5002 (+ 80.4%)	+ 66.6
		Hybrid	7349 (3.0)	11,273 (4.3)	+ 3924 (+ 53.4%)	+ 41.7
		No revasc	49,401 (20.4)	35,009 (13.4)	− 14,392 (− 29.1%)	− 34.5
	III/IV	ev	77,912 (29.2)	111,639 (37.6)	+ 33,727 (+ 43.3%)	+ 28.9
		Surgical	56,958 (21.4)	46,023 (15.5)	− 10,935 (− 19.2%)	− 27.3
		Two-step	10,325 (3.9)	15,801 (5.3)	+ 5476 (+ 53.0%)	+ 37.6
		Hybrid	6644 (2.5)	10,869 (3.7)	+ 4225 (+ 63.6%)	+ 47.1
		No revasc	114,860 (43.1)	112,223 (37.8)	− 2637 (− 2.3%)	− 12.1
Age, years (mean)	IIb	ev	67.1 ± 10.3	68.1 ± 10.0	+ 1.0 (+ 1.6%)	
		Surgical	66.4 ± 9.8	67.5 ± 9.4	+ 1.1 (+ 1.6%)	
		Two-step	66.2 ± 9.8	67.3 ± 9.3	+ 1.1 (+ 1.6%)	
		Hybrid	66.4 ± 9.5	67.4 ± 9.2	+ 0.9 (+ 1.4%)	
		No revasc	68.5 ± 10.9	69.5 ± 10.8	+ 0.9 (+ 1.3%)	
	III/IV	ev	74.1 ± 10.7	75.5 ± 10.6	+ 1.3 (+ 1.8%)	
		Surgical	72.0 ± 10.8	72.1 ± 10.6	+ 0.2 (+ 0.2%)	
		Two-step	71.4 ± 10.7	71.6 ± 10.6	+ 0.2 (+ 0.3%)	
		Hybrid	71.4 ± 10.8	71.5 ± 10.5	+ 0.1 (+ 0.1%)	
		No revasc	75.6 ± 11.2	76.6 ± 11.1	+ 1.0 (+ 1.4%)	
Sex, male	IIb	ev	86,506 (66.7)	105,170 (64.3)	+ 18,664.0 (+ 21.6%)	− 3.7
		Surgical	36,068 (72.8)	29,225 (71.2)	− 6,843.0 (− 19.0%)	− 2.3
		Two-step	4449 (71.5)	7865 (70.1)	+ 3,416.0 (+ 76.8%)	− 2.0
		Hybrid	5285 (71.9)	7929 (70.3)	+ 2,644.0 (+ 50.0%)	− 2.2
		No revasc	33,064 (66.9)	23,010 (65.7)	− 10,054.0 (− 30.4%)	− 1.8
	III/IV	ev	44,914 (57.6)	66,343 (59.4)	+ 21,429.0 (+ 47.7%)	+ 3.1
		Surgical	35,471 (62.3)	29,537 (64.2)	− 5,934.0 (− 16.7%)	+ 3.1
		Two-step	6505 (63.0)	10,094 (63.9)	+ 3,589.0 (+ 55.2%)	+ 1.4
		Hybrid	4171 (62.8)	6906 (63.5)	+ 2,735.0 (+ 65.6%)	+ 1.2
		No revasc	65,955 (57.4)	66,492 (59.2)	+ 537.0 (+ 0.8%)	+ 3.2
In-hospital stay, days (median)	IIb	ev	2 (1–8)	2 (1–3)	+ 0.0 (+ 0.0%)	
		Surgical	10 (8–13)	8 (7–11)	− 2.0 (− 20.0%)	
		Two-step	10 (8–14)	8 (6–11)	− 2.0 (− 20.0%)	
		Hybrid	8 (7–11)	8 (6–10)	+ 0.0 (+ 0.0%)	
		No revasc	3 (1–8)	2 (1–7)	− 1.0 (− 33.3%)	
	III/IV	ev	7 (3–15)	7 (3–13)	+ 0.0 (+ 0.0%)	
		Surgical	17 (12–28)	15 (10–25)	− 2.0 (− 11.8%)	
		Two-step	21 (14–35)	17 (11–30)	− 4.0 (− 19.0%)	
		Hybrid	15 (10–24)	14 (9–23)	− 1.0 (− 6.7%)	
		No revasc	12 (6–20)	10 (5–17)	− 2.0 (− 16.7%)	
Reimbursement, cost per case, € (median)	IIb	ev	2632 (2535–3579)	3161 (2097–3509)	+ 529.0 (+ 20.1%)	
		Surgical	6713 (4396–7004)	7192 (6387–8431)	+ 479.0 (+ 7.1%)	
		Two-step	6025 (4298–6897)	7006 (6120–8408)	+ 981.0 (+ 16.3%)	
		Hybrid	5077 (4275–6665)	6569 (6095–7769)	+ 1,492.0 (+ 29.4%)	
		No revasc	2027 (1961–2098)	2351 (1091–2378)	+ 324.0 (+ 16.0%)	
	III/IV	ev	4429 (4172–6410)	5511 (3174–8235)	+ 1,082.0 (+ 24.4%)	
		Surgical	8901 (6740–12,123)	10,394 (8235–16,746)	+ 1,493.0 (+ 16.8%)	
		Two-step	9961 (6794–12,652)	11,248 (8220–17,168)	+ 1,287.0 (+ 12.9%)	
		Hybrid	8053 (6139–10,805)	8767 (7700–14,155)	+ 714.0 (+ 8.9%)	
		No revasc	4133 (2050–6628)	4110 (2358–6880)	− 23.0 (− 0.6%)	
Van Walraven score (mean, median)	IIb	ev	3.8 ± 3.9	4.3 ± 4.3	+ 0.5 (+ 13.6%)	
			2 (2–5)	2 (2–7)		
		Surgical	4.5 ± 4.7	4.9 ± 5.0	+ 0.5 (+ 10.3%)	
			2 (2–7)	2 (2–7)		
		Two-step	4.5 ± 4.8	5.1 ± 5.2	+ 0.6 (+ 14.0%)	
			2 (2–7)	2 (2–7)		
		Hybrid	4.2 ± 4.5	4.9 ± 5.0	+ 0.6 (+ 15.1%)	
			2 (2–7)	2 (2–7)		
		No revasc	4.9 ± 5.1	5.8 ± 5.6	+ 0.9 (+ 17.4%)	
			2 (2–7)	2 (2–7)		
	III/IV	ev	7.5 ± 6.5	8.6 ± 7.0	+ 1.1 (+ 15.2%)	
			7 (2–12)	7 (2–12)		
		Surgical	8.2 ± 7.0	9.0 ± 7.4	+ 0.8 (+ 9.8%)	
			7 (2–12)	7 (2–13)		
		Two-step	8.6 ± 7.1	9.3 ± 7.6	+ 0.7 (+ 8.4%)	
			7 (2–13)	7 (2–14)		
		Hybrid	7.8 ± 6.9	8.8 ± 7.4	+ 0.9 (+ 12.0%)	
			7 (2–12)	7 (2–13)		
		No revasc	9.0 ± 7.2	10.0 ± 7.6	+ 1.1 (+ 12.0%)	
			7 (2–14)	8 (2–14)		

Data are number (percentage), mean ± standard deviation or median (interquartile range). Font = Fontaine Stage, ev = endovascular

**Table 2:** In-hospital outcome of patients admitted due to PAD and changes from 2009–2011 to 2016–2018

Characteristic	Font	Treatment	2009–2011	2016–2018	Abs. change	Rel. Change %
In-hospital death	IIb	Endovascular	108 (0.1)	123 (0.1)	+ 15 (+ 13.9%)	− 9.7
		Surgical	280 (0.6)	188 (0.5)	− 92 (− 32.9%)	− 19.0
		Two-step	46 (0.7)	71 (0.6)	+ 25 (+ 54.3%)	− 14.4
		Hybrid	32 (0.4)	64 (0.6)	+ 32 (+ 100%)	+ 30.4
		No revasc	263 (0.5)	187 (0.5)	− 76 (− 28.9%)	+ 0.3
	III/IV	Endovascular	2025 (2.6)	2654 (2.4)	+ 629 (+ 31.1%)	− 8.5
		Surgical	3139 (5.5)	2410 (5.2)	− 729 (− 23.2%)	− 5.0
		Two-step	677 (6.6)	959 (6.1)	+ 282 (+ 41.7%)	− 7.4
		Hybrid	341 (5.1)	540 (5.0)	+ 199 (+ 58.4%)	− 3.2
		No revasc	8541 (7.4)	7334 (6.5)	− 1207 (− 14.1%)	− 12.1
Amputations (major)	IIb	Endovascular	8 (0.0)	5 (0.0)	− 3 (− 37.5%)	− 50.5
		Surgical	98 (0.2)	19 (0.0)	− 79 (− 80.6%)	− 76.6
		Two-step	23 (0.4)	27 (0.2)	+ 4 (+ 17.4%)	− 34.9
		Hybrid	< 5	< 5	NA	NA
		No revasc	49 (0.1)	17 (0.0)	− 32 (− 65.3%)	− 51.0
	III/IV	Endovascular	2847 (3.7)	3004 (2.7)	+ 157 (+ 5.5%)	− 26.4
		Surgical	4487 (7.9)	2871 (6.2)	− 1616 (− 36.0%)	− 20.8
		Two-step	1254 (12.1)	1179 (7.5)	− 75 (− 6.0%)	− 38.6
		Hybrid	439 (6.6)	529 (4.9)	+ 90 (+ 20.5%)	− 26.3
		No revasc	19,940 (17.4)	14,288 (12.7)	− 5652 (− 28.3%)	− 26.7
Amputations (minor)	IIb	Endovascular	29 (0.0)	23 (0.0)	− 6 (− 20.7%)	− 37.2
		Surgical	48 (0.1)	32 (0.1)	− 16 (− 33.3%)	− 19.6
		Two-step	10 (0.2)	10 (0.1)	± 0 (± 0%)	− 44.6
		Hybrid	< 5	7 (0.1)	NA	NA
		No revasc	76 (0.2)	36 (0.1)	− 40 (− 52.6%)	− 33.2
	III/IV	Endovascular	12,674 (16.3)	20,236 (18.1)	+ 7562 (+ 59.7%)	+ 11.4
		Surgical	10,945 (19.2)	9741 (21.2)	− 1204 (− 11.0%)	+ 10.1
		Two-step	3090 (29.9)	4592 (29.1)	+ 1502 (+ 48.6%)	− 2.9
		Hybrid	1243 (18.7)	2106 (19.4)	+ 863 (+ 69.4%)	+ 3.6
		No revasc	22,659 (19.7)	23,826 (21.2)	+ 1167 (+ 5.2%)	+ 7.6

Data are number (percentage), mean ± standard deviation or median (interquartile range). Font = Fontaine Stage, NA = data not available

### Type of Treatment

**Fig. 2:**
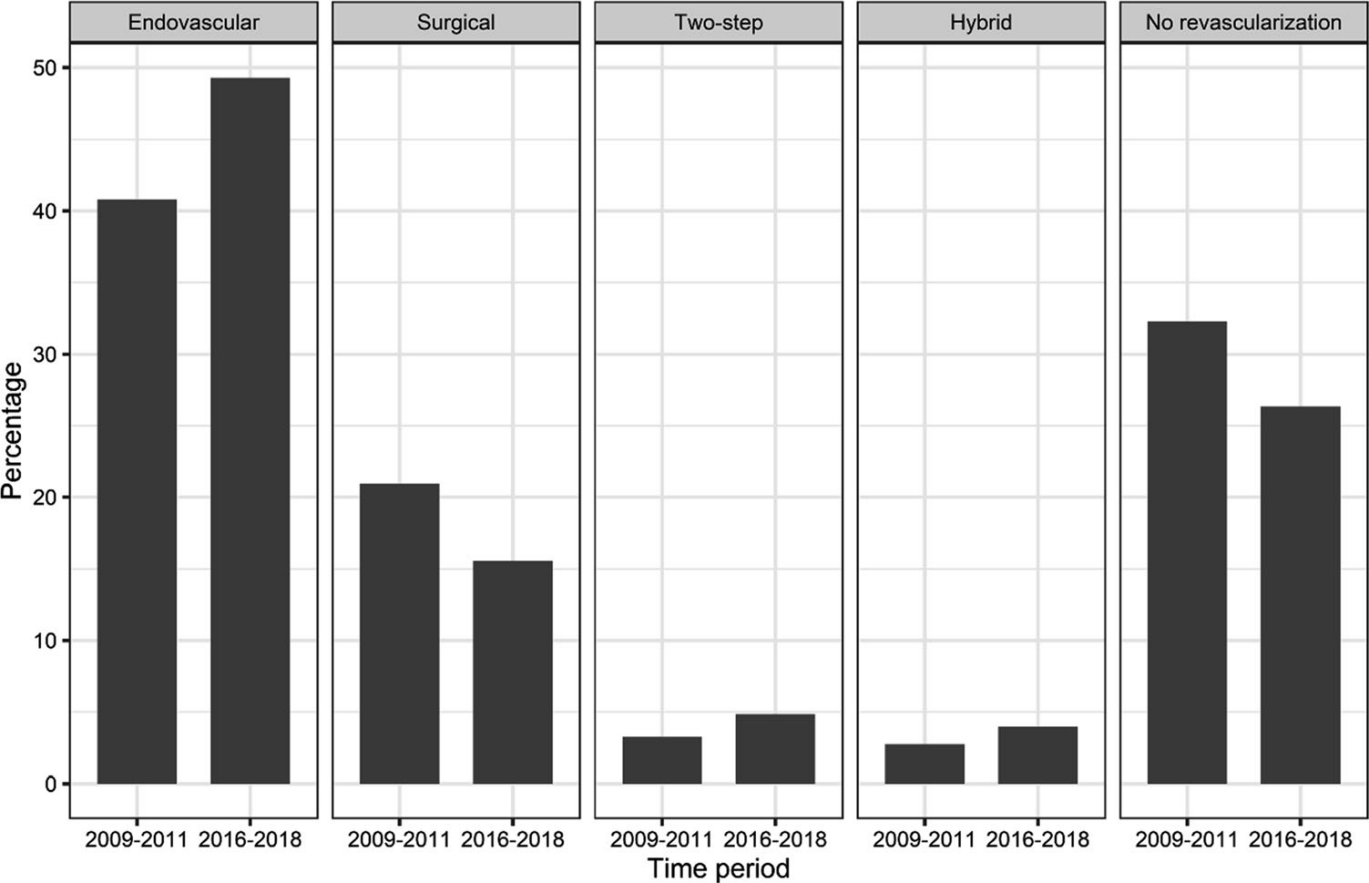
Percentage distribution of different revascularization approaches of patients with peripheral artery disease and their change between the time periods 2009–2011 and 2016–2018

Overall, 70.1% of patients received any type of revascularization during their stay increasing by 8.9% from 67.7% in 2009–2011 to 73.7% in 2016–2018. This increase was mostly driven by a surge of hybrid, two-step and sole endovascular revascularizations, which rose from 40.8 to 49.3%, while sole surgical revascularizations decreased from 20.9 to 15.6% (Fig. 2). The rise of sole endovascular revascularizations was accompanied by a rise of combined two-step or hybrid revascularizations, which increased by 80.4%/53.4% and by 53.0%/63.6% for patients with PAD Fontaine IIb and III/IV, respectively. Endovascular revascularizations were associated with shorter in-hospital stay (median 7 vs. 15 days), lower costs (€5,511 vs. €10,394 per case) and lower in-hospital mortality (2.4 vs. 5.2%) compared to surgical revascularizations exemplarily in patients with PAD Fontaine III/IV in 2016–2018. Comorbidity burden showed only minor differences among different types of treatment being the lowest in hospitalizations with sole endovascular revascularizations (vWs 5.6 ± 5.2) and the highest in those with a two-step approach (vWs 7.3 ± 6.4). Compared to two-step surgical and endovascular approaches during one hospitalization, true hybrid revascularizations in one setting were associated with shorter in-hospital stay (14 vs. 17 days), lower costs (€8,767 vs. € 11,248) and lower in-hospital mortality (5.0 vs. 6.1%) in PAD patients Fontaine III/IV 2016–2018.

### Dichotomization by van Walraven Comorbidity Score (vWs)

**Fig. 3:**
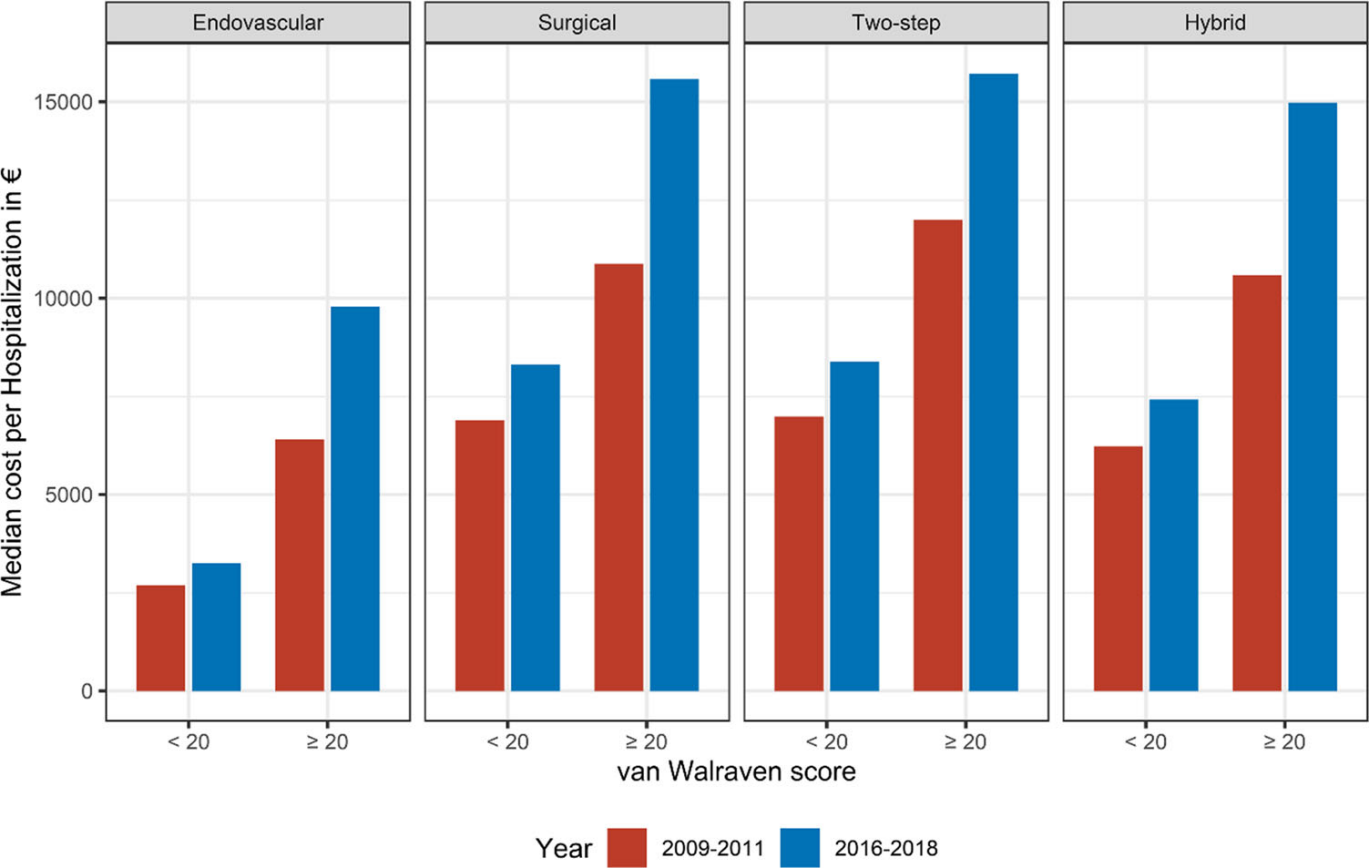
Changes in costs among different revascularization approaches of PAD patients with lower (vWs < 20) and higher comorbidity (vWs ≥ 20) between the time periods 2009–2011 and 2016–2018. vWs = van Walraven score; PAD = peripheral artery disease

As we observed a significant increase in associated cost affecting patients with vWs ≥ 20 (+ 24.6%, + €1,750 per case) between 2009–2011 and 2016–2018, we divided the data in patients with vWs < 20 and ≥ 20 for further analysis to identify factors that might have contributed to this observation (Fig. 3). From 2011 to 2018, the overall case mix index decreased by 0.03 (1.64–1.61) in the subgroup of hospitalizations with vWs < 20 but relevantly increased by 0.19 (2.56–2.75) in hospitalizations of patients with vWs ≥ 20, reflecting a higher case complexity and resulting in higher reimbursement.

Additionally, from 2009–2011 to 2016–2018, hospitalizations of patients with vWs ≥ 20 showed a significant rise of 46.8% (21,444–31,478 cases) with an increase in both major (7.2–7.6%) and minor amputations (16.7–17.3%), an increase of Fontaine IV patients with ulcers (26.3–31.7%) and a rise of endovascular, two-step and hybrid revascularization procedures that increased by 42.8, 44.1 and 58.8%, respectively (Table 3). Ultimately, there was a clear escalation of therapy in these significantly more morbid patients.

**Table 3:** Characteristics of patients admitted due to PAD and changes from 2009–2011 to 2016–2018 dichotomized by comorbidity burden defined by van Walraven score

	vWs	2009–2011	2016–2018	Absolute change	Relative change %
Hospitalizations	All	508,886 (100.0)	558,785 (100.0)	+ 49,899 (+ 9.8%)	
	< 20	487,442 (95.8)	527,307 (94.4)	+ 39,865 (+ 8.2%)	− 1.5
	≥ 20	21,444 (4.2)	31,478 (5.6)	+ 10,034 (+ 46.8%)	+ 33.7
Age, years	< 20	70.6 ± 11.3	71.5 ± 11.1	+ 0.9 (+ 1.3%)	
	≥ 20	76.7 ± 9.4	77.5 ± 9.4	+ 0.7 (+ 0.9%)	
Sex, male	< 20	309,293 (63.5)	332,718 (63.1)	+ 23,425 (+ 7.6%)	− 0.6
	≥ 20	13,095 (61.1)	19,853 (63.1)	+ 6758 (+ 51.6%)	+ 3.3
In-hospital death	< 20	11,593 (2.4)	9695 (1.8)	− 1898 (− 16.4%)	− 22.7
	≥ 20	3859 (18.0)	4835 (15.4)	+ 976 (+ 25.3%)	− 14.6
In-hospital stay, days	< 20	7 (2–14)	6 (2–11)	− 1 (− 14.3%)	
	≥ 20	17 (9–30)	15 (8–27)	− 2 (− 11.8%)	
Reimbursement per case, €	< 20	4244 (2535–6731)	4193 (2379–7241)	− 51.4 (− 1.2%)	
	≥ 20	7105 (4317–11,022)	8855 (5039–13,846)	+ 1749.9 (+ 24.6%)	
Total reimbursement, million €	< 20	2,519 (92.5)	3,119 (89.5)	+ 600.0 (+ 23.8%)	− 3.3
	≥ 20	203 (7.5)	367 (10.5)	+ 163.6 (+ 80.5%)	+ 41.0
Major amputations	< 20	22,425 (4.6)	16,352 (3.1)	− 6073 (− 27.1%)	− 32.6
	≥ 20	3447 (16.1)	3556 (16.6)	+ 109 (+ 3.2%)	+ 3.2
Minor amputations	< 20 ≥ 20	35,309 (7.2)	40,087 (7.6)	+ 4778 (+ 13.5%)	+ 4.9
		3588 (16.7)	5443 (17.3)	+ 1855 (+ 51.7%)	+ 3.3
PAD stage					
IIb	< 20	239,804 (49.2)	258,645 (49.1)	+ 18,841 (+ 7.9%)	− 0.3
III		66,164 (13.6)	65,267 (12.4)	− 897 (− 1.4%)	− 8.8
IVu		79,657 (16.3)	104,607 (19.8)	+ 24,950 (+ 31.3%)	+ 21.4
IVg		101,817 (20.9)	98,788 (18.7)	− 3029 (− 3.0%)	− 10.3
IIb	≥ 20	2383 (11.1)	3585 (11.4)	+ 1202 (+ 50.4%)	+ 2.5
III		2775 (12.9)	3487 (11.1)	+ 712 (+ 25.7%)	− 14.4
IVu		5645 (26.3)	9989 (31.7)	+ 4344 (+ 77.0%)	+ 20.5
IVg		10,641 (49.6)	14,417 (45.8)	+ 3776 (+ 35.5%)	− 7.7
Type of therapy					
endovascular	< 20	202,956 (41.6)	265,552 (50.4)	+ 62,596 (+ 8.7%)	+ 21.0
surgical		101,650 (20.9)	81,989 (15.5)	− 19,661 (− 5.3%)	− 25.4
two-step		15,640 (3.2)	25,106 (4.8)	+ 9466 (+ 1.6%)	+ 48.4
hybrid		13,471 (2.8)	20,925 (4.0)	+ 7454 (+ 1.2%)	+ 43.6%
no revasc		153,725 (31.5)	133,735 (25.4)	− 19,990 (− 6.2%)	− 19.6
Endovascular	≥ 20	4653 (21.7)	9755 (31.0)	+ 5102 (+ 9.3%)	+ 42.8
surgical		4825 (22.5)	5089 (16.2)	+ 264 (− 6.3%)	− 28.1
two-step		908 (4.2)	1920 (6.1)	+ 1012 (+ 1.9%)	+ 44.1
hybrid		522 (2.4)	1217 (3.9)	+ 695 (+ 1.4%)	+ 58.8
no revasc		10,536 (49.1)	13,497 (42.9)	+ 2961 (− 6.3%)	− 12.7

Data are number (percentage), mean ± standard deviation or median (interquartile range). vWs = van Walraven score

### Performed Revascularization Procedures

Between 2009–2011 and 2016–2018, the strongest increase in revascularization procedures was observed in hybrid and sole endovascular revascularization with an absolute increase of 129.1% (39,301–90,019) and 63.3% (381,694–623,439). Among all types of hospitalizations with revascularizations, the absolute number of procedures in patients with higher comorbidity (vWs ≥ 20) increased up to twofold more compared to patients with vWs < 20 (overall + 90.6% vs. + 41.5%). Consequently, subgroups of more comorbid patients showed a relative increase in performed hybrid (+ 71.6%), two-step (+ 32.6%) and sole endovascular procedures (+ 28.5%) but showed a 39.2% decrease in sole surgical approaches (Table 4). Among individual endovascular procedures, we observed a strong increase in the use of drug-eluting stents and drug-coated balloons, and conductions of endovascular thrombectomy and rotational thrombectomy (Supplemental Table 1, 3, 4). For surgical interventions, the number of patch plastics and endarterectomies increased, while bypass procedures and embolectomies decreased (Supplemental Table 2, 3, 4).

**Table 4:** Changes in individual revascularization procedures for each type of revascularization

	2009–2011		2016–2018		Absolute change		Relative change	
	vWs < 20	vWS ≥ 20	vWs < 20	vWS ≥ 20	vWs < 20	vWS ≥ 20	vWs < 20%	vWS ≥ 20%
Total	736,604 (100)	27,760	1,042,056	52,904	+ 305,452 (+ 41.5%)	+ 25,144 (+ 90.6%)		
Endovascular	372,555 (50.6)	9139 (32.9)	601,061 (57.7)	22,378 (42.3)	+ 228,506 (+ 61.3%)	+ 13,239 (+ 144.9%)	+ 14.0	+ 28.5
Surgical	257,756 (35.0)	12,981 (46.8)	230,783 (22.1)	15,044 (28.4)	− 26,973 (− 10.5%)	+ 2063 (+ 15.9%)	− 36.7	− 39.2
Two-step	68,733 (9.3)	3989 (14.4)	125,593 (12.1)	10,082 (19.1)	+ 56,860 (+ 82.7%)	+ 6093 (+ 152.7%)	+ 29.1	+ 32.6
Hybrid	37,560 (5.1)	1651 (5.9)	84,619 (8.1)	5400 (10.2)	+ 47,059 (+ 125.3%)	+ 3749 (+ 227.1%)	+ 59.3	+ 71.6

Data are number (percentage). Multiple individual revascularizations can be performed during one hospitalization

## Discussion

This decade-long nationwide study analyzed the characteristics, trends, costs, and outcomes of different revascularization approaches in patients hospitalized due to PAD between 2009–2011 and 2016–2018. The most important results of this study are: (1) the proportion of hospitalizations with any type of revascularization procedure increased; (2) sole endovascular, two-step and hybrid interventions increased, while sole surgical approaches decreased; (3) hospitalizations with sole endovascular procedures were associated with lower costs, shorter in-hospital stay, and lower in-hospital mortality compared to any other revascularization approach; (4) the proportion of more comorbid patients rose and is associated with an overproportionate increase in individual revascularization procedures and accompanying reimbursement costs.

In an aging society, the number of patients with PAD is increasing and PAD-related healthcare costs are rising along with it. Over the past decades, a shift from surgical to endovascular revascularization approaches has been well recognized and is associated with lower individual procedural costs and lower procedural risk but comparable to improved outcomes [4, 9, 23–25]. Apart from sole endovascular or surgical revascularization approaches, joint hybrid interventions are increasingly performed showing less complications, lower mortality and shorter hospitalization compared to open surgical revascularizations [16, 17, 26, 27].

In this study, we identified several reasons that contribute to the rise in reimbursement costs. First, as several previous studies have shown we likewise found an increasing number of patients with older age and more advanced stages of PAD [13, 28]. Second, the number of patients with higher comorbidity (that also showed the strongest increase in reimbursement costs) increased by over one third. Third, the absolute number of individual revascularization procedures increased in all but the sole surgical hospitalizations.

When we analyzed the respective reimbursement costs for patients with increasing comorbidity scores, we observed a significant surge of costs for those with higher comorbidity. The main reason for this observation seems to be the increase of the absolute and relative number of more morbid patients and the higher rate of treatment strategies including any type of revascularization and especially both joint or two-step surgical and endovascular approaches, rather than none. These findings are in line with a recent study by Fereydooni et al. that reported an increasing use of hybrid revascularizations between 2010 and 2017 in the Vascular Quality Initiative database [16]. Compared to other recent studies that analyzed hybrid revascularization approaches, we additionally divided this group into subgroups (hybrid or two-step) according to whether these interventions were performed as a real hybrid revascularization in one setting or if both an endovascular and surgical revascularizations were performed in one hospitalization [16–18]. In addition to lower costs and shorter stay, we also found lower endovascular procedural codes in the true hybrid group. In a large number of the operations marked as hybrid procedures by means of an additional hybrid OPS code, no additional interventional code, e.g., for a PTA or stent implantation, was coded. This may be explained by the fact that usually, the additional coding of interventions does not result in an increase in reimbursement and is therefore possibly coded carelessly. In contrast, in hospitalizations with two separate endovascular and surgical procedures, each approach is encoded separately.

While endovascular revascularization procedures are rising, in-hospital mortality and major amputations of the lower extremity are decreasing [4, 13, 29, 30]. Although in our study, major amputations declined in all PAD stages and revascularization approaches between 2009–2011 and 2016–2018, both major and minor amputations showed an increase in the subgroup of patients with high comorbidity. This contrasting observation might be due to an escalation of therapy options in this particularly affected cohort as the associated in-hospital mortality declined over the observed time period. It is important to note that in-hospital mortality declined among all subgroups which indicates a success of the therapy escalation and may justify higher costs.

Overall, it has to be recognized that especially the number of PAD patients with higher comorbidity is increasing and leading to a disproportional increase in associated costs due to an increasing number of individually performed revascularization procedures which are leading to more complex hospitalizations but less in-hospital mortality.

Apart from its evident strengths due to the large real-world dataset of a representative healthcare system, this study also has some limitations. First, using the data provided we were only able to include in-patient treatments and no ambulatory cases. Although studies from other healthcare systems demonstrated that the ambulatory PAD treatment is practicable, revascularization procedures in Germany are almost exclusively performed in hospitals [31, 32]. Second, we present data from individual hospitalizations and individual patients cannot be identified. This generates a bias due to the multiple occurrences of patients that had multiple hospitalizations which does not allow a simple generalization of our results to patients with PAD in general. Third, using in-hospital data we were able to only identify in-hospital mortality and amputations as primary outcome. Differences in general mortality, readmission and re-revascularization rates among different revascularization approaches could not be assessed by these data. Fourth, reimbursement does not exactly reflect the actual costs of an individual hospital stay, but rather the average costs assigned to a given DRG across Germany. Last, we used administrative data that was collected for remuneration purposes by each treating hospital. Errors in coding practices and economic motivations might add a bias that cannot be avoided or identified.

In conclusion, this study shows a rise of hybrid, two-step, and sole endovascular revascularization approaches for PAD, while sole surgical interventions are decreasing. An increasing number of PAD patients with high comorbidity leads to a surge in associated reimbursement costs, which are additionally increasing due to a rising number of individually performed revascularization procedures. While this escalation of therapy overall leads to lower associated in-hospital mortality, it is to be expected that healthcare costs for the treatment of PAD will continue to rise with more patients that also show a higher comorbidity and more advanced stages of the disease.

## Supplementary Information

Below is the link to the electronic supplementary material.

Supplementary file1 (DOCX 58 KB)
