# Supplementary material for: Comparison and Trends of Endovascular, Surgical and Hybrid Revascularizations and the Influence of Comorbidity in 1 Million Hospitalizations Due to Peripheral Artery Disease in Germany Between 2009 and 2018
Source: Cardiovasc Intervent Radiol. 2022 Apr 15;45(10):1472–82. doi: 10.1007/s00270-022-03136-9 (PMC9499912; doi:10.1007/s00270-022-03136-9)
Supplement: Supplementary file 1 — Supplementary file1 (DOCX 58 KB) [file 270_2022_3136_MOESM1_ESM.docx]

**Inclusion procedures - endovascular revascularization:**

- Balloon angioplasty
- Blade angioplasty
- Endovascular atherectomy
- Laser angioplasty
- Rotational thrombectomy
- Selective thrombolysis
- Drug coated balloon
- Bare metal stent
- Drug eluting stent
- Stent graft

*Endovascular OPS-Codes:*

88360b, 88360c, 883609, 88360e, 883bb0, 883bb2, 883bb3, 883bb4, 883bb5, 88361b, 88361c, 883619, 88361e, 884009, 88400b, 88400c, 884019, 88401b, 88401c, 884029, 88402b, 88402c, 884039, 88403b, 88403c, 884049, 88404b, 88404c, 884059, 88405b, 88405c, 88400e, 88401e, 88402e, 88403e, 88404e, 88405e, 884109, 88410b, 88410c, 884119, 88411b, 88411c, 884129, 88412b, 88412c, 884139, 88413b, 88413c, 884149, 88414b, 88414c, 884159, 88415b, 88415c, 88410e, 88411e, 88412e, 88413e, 88414e, 88415e, 884209, 88420b, 88420c, 884219, 88421b, 88421c, 884229, 88422b, 88422c, 884239, 88423b, 88423c, 884249, 88424b, 88424c, 884259, 88425b, 88425c, 88420e, 88421e, 88422e, 88423e, 88424e, 88425e, 884909, 88490b, 88490c, 884919, 88491b, 88491c, 884929, 88492b, 88492c, 884939, 88493b, 88493c, 884949, 88494b, 88494c, 884959, 88495b, 88495c, 88490e, 88491e, 88492e, 88493e, 88494e, 88495e, 88363b, 88363c, 883639, 88363e, 88362b, 88362c, 883629, 88362e, 88368b, 88368c, 883689, 88368e, 88367b, 88367c, 883679, 88367e, 8836pb, 8836pc, 8836p9, 8836pe, 88360b, 88360s, 88360c, 883609, 88360q, 88360e, 883bba, 883bbb, 883bbc, 883bbd, 88361b, 88361k, 88361c, 88361h, 883619, 88361e, 884009, 88400q, 88400b, 88400s, 88400c, 884019, 88401q, 88401b, 88401s, 88401c, 884029, 88402q, 88402b, 88402s, 88402c, 884039, 88403q, 88403b, 88403s, 88403c, 884049, 88404q, 88404b, 88404s, 88404c, 884059, 88405q, 88405b, 88405s, 88405c, 88400e, 88401e, 88402e, 88403e, 88404e, 88405e, 884109, 88410q, 88410b, 88410s, 88410c, 884119, 88411q, 88411b, 88411s, 88411c, 884129, 88412q, 88412b, 88412s, 88412c, 884139, 88413q, 88413b, 88413s, 88413c, 884149, 88414q, 88414b, 88414s, 88414c, 884159, 88415q, 88415b, 88415s, 88415c, 88410e, 88411e, 88412e, 88413e, 88414e, 88415e, 884209, 88420q, 88420b, 88420s, 88420c, 884219, 88421q, 88421b, 88421s, 88421c, 884229, 88422q, 88422b, 88422s, 88422c, 884239, 88423q, 88423b, 88423s, 88423c, 884249, 88424q, 88424b, 88424s, 88424c, 884259, 88425q, 88425b, 88425s, 88425c, 88420e, 88421e, 88422e, 88423e, 88424e, 88425e, 884909, 88490q, 88490b, 88490s, 88490c, 884919, 88491q, 88491b, 88491s, 88491c, 884929, 88492q, 88492b, 88492s, 88492c, 884939, 88493q, 88493b, 88493s, 88493c, 884949, 88494q, 88494b, 88494s, 88494c, 884959, 88495q, 88495b, 88495s, 88495c, 88490e, 88491e, 88492e, 88493e, 88494e, 88495e, 88363b, 88363k, 88363c, 88363h, 883639, 88363e, 88362b, 88362k, 88362c, 88362h, 883629, 88362e, 88368b, 88368k, 88368c, 88368h, 883689, 88368e, 88367b, 88367k, 88367c, 88367h, 883679, 88367e, 8836pb, 8836pk, 8836pc, 8836ph, 8836p9, 8836pe

**Inclusion procedures - surgical revascularization:**

- Endatherectomy
- Patch plastic
- Embolectomy/Thrombectomy
- Bypass

*Surgical OPS-Codes:*

538152, 538153, 538154, 538155, 538156, 53815x, 538170, 538171, 538172, 538173, 53817x, 538180, 538182, 538183, 538184, 538187, 53818x, 539552, 539553, 539554, 539555, 539552, 53955x, 539570, 539571, 539572, 539573, 53957x, 539580, 539582, 539583, 539584, 539587, 53958x, 538051, 538052, 538053, 538054, 538055, 538056, 53805x, 538070, 538071, 538072, 538073, 53807x, 538080, 538081, 538082, 538083, 538084, 538085, 538086, 538087, 53808x, 539341, 539342, 539343, 539344, 539345, 539346, 539347, 539351, 539352, 539353, 539354, 539355, 539356, 539357, 53935x, 539361, 539362, 53936x

**Codes for hybrid (joint endovascular and surgical) revascularization procedures:**

598a0, 538c09, 538c0b, 538c0c, 538c0e, 538c19, 538c1b, 538c1c, 538c1e, 538c29, 538c2b, 538c2c, 538c2e, 538d09, 538d0b, 538d0c, 538d0e, 538d19, 538d1b, 538d1c, 538d1e, 538d29, 538d2b, 538d2c, 538d2e, 538e09, 538e0b, 538e0c, 538e0e, 538e19, 538e1b, 538e1c, 538e1e, 538e29, 538e2b, 538e2c, 538e2e, 538f9, 538fb, 538fc

**Definition of revascularization types:**

**None**: No endovascular revascularization procedure

**Endovascular**: Only endovascular revascularization

**Surgical**: Only surgical revascularization

**Hybrid**: Endovascular and surgical revascularization with code for hybrid approach.

**Two-step**: Endovascular and surgical revascularization but absence of hybrid codes

**Following are detailed tables for individual revascularization procedures in each type of revascularization. It must be noted, that only in the two-step group, both a surgical and endovascular code had to be present. The hybrid group was defined by the presence of a special hybrid-code, that can only be assigned when a joint endovascular and surgical revascularization was performed in one single intervention.**

| **Supplemental Table 1**  Endovascular revascularization procedures with regard to their anatomical location and dichotomized by comorbidity burden defined by *van Walraven score* | | | | | | | | | |
| --- | --- | --- | --- | --- | --- | --- | --- | --- | --- |
|  |  | **2009 - 2011** | | **2016 - 2018** | | **Absolute change** | | **Relative change** | |
|  |  | **vWs < 20** | **vWS ≥ 20** | **vWs < 20** | **vWS ≥ 20** | **vWs < 20** | **vWS ≥ 20** | **vWs < 20** | **vWS ≥ 20** |
| **All endovascular revascularization procedures** |  | 372,555 | 9,139 | 601,061 | 22,378 | +228,506 | 13,239 | **+61.3%** | **+144.9%** |
| **Angioplasty** | total  aorto-iliac  upper leg  below the knee  bypass graft | 239,527 (64.3)  55,189 (14.8)  128,923 (34.6)  54,535 (14.6)  880 (0.2) | 6,428 (70.3)  702 (7.7)  3,075 (33.6)  2,618 (28.6)  0 (0.0) | 329,557 (54.8)  69,017 (11.5)  174,607 (29.1)  83,738 (13.9)  2,195 (0.4) | 14,005 (62.6)  1,362 (6.1)  6,398 (28.6)  6,159 (27.5)  86 (0.4) | +90,030 (-9.5)  +13,828 (-3.3)  +45,684 (-5.6)  +29,203 (-0.7)  +1,315 (+0.1) | +7,577 (-7.8)  +660 (-1.6)  +3,323 (-5.1)  +3,541 (-1.1)  +86 (+0.4) | **-14.7%**  **-22,.5%**  **-16,.1%**  **-4.8%**  **+54.6%** | **-11.0%**  **-20.8%**  **-15.0%**  **-3.9%**  **n/a** |
| **Drug-coated balloons** |  | 6,072 (1.6) | 125 (1.4) | 81,204 (13.5) | 2,467 (11.0) | +75,132 (+11.9) | +2342 (+9.7) | **+728.9%** | **+706.0%** |
| **Bare metal stent** | total  aorto-iliac  upper leg  below the knee  bypass graft | 100,225 (26.9)  46,209 (12.4)  48,311 (12.9)  5,487 (1.5)  218 (0.06) | 1,772 (19.4)  562 (6.1)  977 (10.7)  224 (2.5)  9 (0.1) | 132,962 (22.1)  59,603 (9.9)  67,464 (11.2)  5,579 (0.9)  316 (0.05) | 3,601 (16.1)  1,140 (5.1)  2,120 (9.5)  330 (1.5)  11 (0.05) | +32,737 (-4.8)  +13,394 (-2.5)  +19,153 (-1.7)  +92 (-0.5)  +98 (-0.006) | +1,829 (-3.3)  +578 (-1.1)  +1,143 (-1.2)  +106 (-1.0)  +2 (-0.05) | **-17.8%**  **-20.1%**  **-13.4%**  **-36.9%**  **-10.2%** | **-17.0%**  **-17.2%**  **-11.4%**  **-39.8%**  **-50.1%** |
| **Drug-eluting stent** | total  aorto-iliac  upper leg  below the knee  bypass graft | 2,879 (0.8)  308 (0.08)  1,271 (0.3)  1,300 (0.3)  0 (0.0) | 78 (0.9)  0 (0.0)  27 (0.3)  51 0.6)  0 (0.0) | 9,924 (1.7)  699 (0.1)  4,740 (0.8)  4,455 (0.7)  30 (0.005) | 407 (1.8)  7 (0.03)  144 (0.6)  188 (0.8)  0 (0.0) | +7,045 (+0.9)  +391 (+0.03)  +3,469 (+0.4)  +3,155 (+0.4)  +30 (+0.005) | +329 (+0.9)  +7 (+0.03)  +117 (+0.3)  +137 (+0.3)  0 (0.0) | **+113.7%**  **+40.7%**  **+131.2%**  **+112.4%**  **n/a** | **+113.1%**  **n/a**  **+117.8%**  **+50.5%**  **n/a** |
| **Stent graft** | total  aorto-iliac  upper leg  below the knee  bypass graft | 2,054 (0.6)  615 (0.2)  1,251 (0.3)  173 (0.05)  15 (0.004) | 29 (0.3)  6 (0.07)  17 (0.2)  6 (0.07)  0 (0.0) | 5,460 (0.9)  2,086 (0.3)  3,004 (0.5)  308 (0.05)  62 (0.01) | 181 (0.8)  52 (0.2)  109 (0.5)  20 (0.09)  0 (0.0) | 3406 (+0.4)  1471 (+0.2)  1753 (+0.2)  135 (+0.004)  47 (+0.006) | +152 (+0.5)  +46 (+0.2)  +173 (+0.3)  +14 (+0.02)  0 (0.0) | **+64.8%**  **+110.2%**  **+48.8%**  **+10.4%**  **+156.2%** | **+154.9**  **+253.9**  **+161.9**  **+36.1**  **n/a** |
| **Atherectomy** | total  aorto-iliac  upper leg  below the knee  bypass graft | 2,019 (0.5)  64 (0.02)  1,643 (0.4)  306 (0.08)  6 (0.002) | 26 (0.3)  0 (0.0)  19 (0.2)  7 (0.08)  0 (0.0) | 1,353 (0.2)  321 (0.05)  1,032 (0.2)  0 (0.0)  0 (0.0) | 45 (0.2)  0 (0.0)  23 (0.1)  22 (0.1)  0 (0.0) | -666 (-0.3)  257 (+0.03)  -611 (-0.3)  -306 (-0.08)  -6 (-0.002) | +19 (-0.8)  0 (0.0)  +4 (-0.1)  +15 (+0.02)  0 (0.0) | **-58.5%**  **+210.9%**  **-61.1%**  **n/a**  **n/a** | **-29.3%**  **n/a**  **-50.6%**  **+28.4%**  **n/a** |
| **Thrombectomy** | total  aorto-iliac  upper leg  below the knee  bypass graft | 4,464 (1.2)  132 (0.04)  1,955 (0.5)  2,303 (0.6)  74 (0.02) | 168 (1.8)  0 (0.0)  79 (0.9)  84 (0.9)  5 (0.05) | 7,678 (1.3)  240 (0.04)  3,278 (0.5)  4,025 (0.7)  135 (0.02) | 407 (1.8)  7 (0.03)  168 (0.8)  227 (1.0)  5 (0.02) | +3,214 (+0.08)  +108 (0.004)  +1,323 (0.02)  +1,722 (0.05)  +61 (0.003) | +239 (-0.02)  +7 (+0.03)  +89 (-0.1)  +143 (+0.09)  0 (-0.03) | **+6.6%**  **+12.7%**  **+3.9%**  **+8.3%**  **+13.1%** | **-1.1%**  **n/a**  **-13.2%**  **+10.4%**  **-59.2%** |
| **Laser angioplasty** | total  aorto-iliac  upper leg  below the knee  bypass graft | 755 (0.2)  30 (0.008)  557 (0.2)  168 (0.05)  0 (0.0) | 11 (0.1)  0 (0.0)  5 (0.05)  6 (0.07)  0 (0.0) | 941 (0.2)  30 (0.005)  658 (0.1)  248 (0.04)  5 (0.0008) | 46 (0.2)  0 (0.0)  20 (0.09)  26 (0.1)  0 (0.0) | +186 (-0.05)  0 (-0.003)  +101 (-0.04)  +80 (-0.04)  +5 (+0.008) | +35 (+0.09)  0 (0.0)  +15 (+0.03)  +20 (+0.05)  0 (0.0) | **-22.7%**  **-38.0%**  **-26.8%**  **-8.5%**  **n/a** | **+70.8%**  **n/a**  **+63.4%**  **+76.9%**  **n/a** |
| **Thrombolysis** | total  aorto-iliac  upper leg  below the knee  bypass graft | 11,078 (3.0)  524 (0.1)  5,959 (1.6)  4,100 (1.1)  495 (0.1) | 399 (4.4)  8 (0.09)  203 (2.2)  174 (1.9)  14 (0.2) | 14,977 (2.5)  662 (0.1)  7,783 (1.3)  5,997 (1.0)  535 (0.09) | 671 (3.0)  19 (0.9)  274 (1.2)  350 (1.6)  28 (0.1) | +3,899 (-0.5)  +138 (-0.03)  +1,824 (-0.3)  +1,897 (-0.1)  +40 (-0.04) | +272 (-1.4)  +11 (-0.002)  +71 (-1.0)  +176 (-0.3)  +14 (-0.03) | **-16.2%**  **-21.7%**  **-19.0%**  **-9.3%**  **-33.0%** | **-31.3%**  **-3.0%**  **-44.9%**  **-17.9%**  **-18.3%** |
| **Rotational thrombectomy** | total  aorto-iliac  upper leg  below the knee  bypass graft | 2,160 (0.6)  163 (0.04)  1,556 (0.4)  363 (0.09)  81 (0.02) | 49 (0.5)  0 (0.0)  40 (0.4)  9 (0.1)  0 (0.0) | 14,597 (2.4)  1,046 (0.2)  11,287 (1.9)  1,957 (0.3)  307 (0.05) | 437 (1.9)  21 (0.09)  323 (1.4)  80 (0.4)  13 (0.06) | +12,437 (+1.8)  +883 (+0.1)  +9,731 (+1.4)  +1,594 (+0.2)  +226 (0.03) | +388 (+1.4)  +21 (+0.1)  +283 (+1.0)  +71 (+0.3)  +13 (+0.06) | **+318.9%**  **+297.8%**  **+349.6%**  **+234.2%**  **+134.9%** | **+264.2%**  **n/a**  **+229.8%**  **+263.0%**  **n/a** |
| **Reentry Device** |  | 910 (0.2) | 26 (0.3) | 2,090 (0.4) | 83 (0.4) | +1180 (0.1) | +57 (+0.09) | **+42.4%** | **+30.4%** |
| **Transplant** | total  autogenic  allogenic  xenogenic  alloplastic | 412 (0.1)  138 (0.04)  0 (0.0)  20 (0.005)  254 (0.07) | 28 (0.3)  6 (0.07)  0 (0.0)  0 (0.0)  22 (0.2) | 318 (0.05)  110 (0.02)  0 (0.0)  59 (0.01)  149 (0.02) | 28 (0.1)  6 (0.03)  0 (0.0)  8 (0.04)  14 (0.06) | -94 (-0.06)  -28 (-0.02)  0 (0.0)  +39 (+0.004)  -105 (-0.04) | 0 (-0.2)  0 (-0.04)  0 (0.0)  +8 (+0.04)  -8 (-0.2) | **-52.2%**  **-50.6%**  **n/a**  **+82.8%**  **-63.6%** | **-59.2%**  **-59.2%**  **n/a**  **n/a**  **-74.0%** |

| **Supplemental Table 2**  Surgical interventions with regard to their anatomical location and dichotomized by comorbidity burden defined by *van Walraven score* | | | | | | | | | | | | | | | | | | | | |
| --- | --- | --- | --- | --- | --- | --- | --- | --- | --- | --- | --- | --- | --- | --- | --- | --- | --- | --- | --- | --- |
|  | |  | | **2009 - 2011** | | | | **2016 - 2018** | | | | **Absolute change** | | | | **Relative change** | | | | |
|  | |  | | **vWs < 20** | | **vWS ≥ 20** | | **vWs < 20** | | **vWS ≥ 20** | | **vWs < 20** | | **vWS ≥ 20** | | **vWs < 20** | | **vWS ≥ 20** | | |
| **All surgical procedures** | |  | | **257,756** | | **12,981** | | **230,783** | | **15,044** | | **-26,973** | | **+2,063** | | **-10.5%** | | **+15.9%** | | |
| **Endarterectomy** | | total  iliac  femoral  deep femoral  popliteal  lower leg  bypass graft | | 80,310 (31.2)  16,507 (6.4)  38,757 (15.0)  18,536 (7.2)  4,089 (1.6)  2,218 (0.9)  203 (0.1) | | 3,296 (25.4)  587 (4.5)  1,548 (11.9)  794 (6.1)  222 (1.7)  145 (1.1)  0 (0.0) | | 84,743 (36.7)  16,331 (7.1)  40,701 (17.6)  20,898 (9.1)  3,736 (1.6)  2,626 (1.1)  451 (0.2) | | 4,769 (31.7)  772 (5.1)  2,235 (14.9)  1,218 (8.1)  266 (1.8)  251 (1.7)  27 (0.2) | | +4,433 (+5.5)  -176 (+0.7)  +1,944 (+2.6)  +2,362 (+1.9)  -353 (+0.03)  +408 (+0.3)  +248 (+0.1) | | +1,473 (+6.3)  +185 (+0.6)  +687 (+2.9)  +424 (+2.0)  +44 (+0.06)  +106 (+0.6)  +27 (+0.2) | | **+17.9%**  **+10.5%**  **+17.3%**  **+25.9%**  **+2.0%**  **+32.2%**  **+148.1%** | | **+24.8%**  **+13.5%**  **+24.6%**  **+32.4%**  **+3.4%**  **+49.4%**  **n/a** | | |
| **Patch plastic** | | total  femoral  deep femoral  popliteal  lower leg  bypass graft | | 22,050 (8.6)  11,911(4.6)  7,049 (2.7)  1,630 (0.6)  1,345 (0.5)  115 (0.04) | | 921 (7.1)  441 (3.4)  312 (2.4)  79 (0.6)  84 (0.6)  5 (0.04) | | 20,627 (8.9)  12,011 (5.2)  5,168 (2.2)  1,310 (0.6)  1,585 (0.7)  553 (0.2) | | 1,273 (8.5)  654 (4.3)  354 (2.4)  97 (0.6)  142 (0.9)  26 (0.2) | | -1,423 (+0.4)  +100 (+0.6)  -1,881 (-0.5)  -320 (-0.06)  +240 (+0.2)  +438 (+0.2) | | +352 (+1.4)  +213 (+0.9)  +42 (-0.05)  +18 (+0.03)  +58 (+0.3)  +21 (+0.1) | | **+4.5%**  **+12.6%**  **-18.1%**  **-10.2%**  **+31.6%**  **+437.1%** | | **+19.3%**  **+28.0%**  **-2.1%**  **+5.9%**  **+45.9%**  **+348.7%** | | |
| **Embolectomy / Thrombectomy** | | total  iliac  femoral  deep femoral  popliteal  lower leg  bypass graft | | 30,280 (11.7)  7,897 (3.1)  7,507 (2.9)  2,102 (0.8)  4,181 (1.6)  6,164 (2.4)  2,429 (0.9) | | 2,203 (17.0)  467 (3.6)  576 (4.4)  220 (1.7)  312 (2.4)  479 (3.7)  149 (1.1) | | 26,483 (11.5)  5,819 (2.5)  6,453 (2.8)  2,222 (0.9)  3,213 (1.4)  5,928 (2.6)  2,848 (1.2) | | 2,058 (13.7)  358 (2.4)  506 (3.4)  203 (1.3)  253 (1.7)  515 (3.4)  223 (1.5) | | -3,797 (-0.3)  -2,078 (-0.5)  -1,054 (-0.1)  +120 (+0.1)  -968 (-0.2)  -236 (+0.2)  +419 (+0.3) | | -145 (-3.3)  -109 (-1.2)  -70 (-1.0)  -17 (-0.3)  -59 (-0.7)  +36 (-0.3)  +74 (+0.3) | | **-2.3%**  **-17.7%**  **-4.0%**  **+18.1%**  **-14.2%**  **+7.4%**  **+30.9%** | | **-19.4%**  **-33.9%**  **-24.2%**  **-20.4%**  **-30.0%**  **-7.2%**  **+29.1%** | | |
| **Bypass** | | total  iliac origin  femoral origin  popliteal origin  upper leg target  lower leg target | | 63,073 (24.5)  7,134 (2.8)  53,254 (20.7)  2,685 (1.0)  35,083 (13.6)  27,990 (10.9) | | 3,497 (26.9)  333 (2.6)  2,948 (22.7)  216 (1.7)  1,528 (11.8)  1,969 (15.2) | | 45,541 (19.7)  4,397 (1.9)  39,066 (16.9)  2,078 (0.9)  23,935 (10.4)  21,606 (9.4) | | 3,426 (22.8)  256 (1.7)  2,922 (19.4)  248 (1.6)  1327 (8.8)  2,099 (14.0) | | -17,532 (-4.7)  -2,737 (-0.9)  -14,188 (-3.7)  -607 (-0.1)  -11,148 (-3.2)  -6,384 (-1.5) | | -71 (-4.1)  -77 (-0.9)  -26 (-3.3)  +32 (-0.02)  -201 (-3.0)  130 (-1.2) | | **-19.4%**  **-31.2%**  **-18.1%**  **-13.6%**  **-23.8%**  **-13.8%** | | **-15.5%**  **-33.7%**  **-14.5%**  **-0.9%**  **-25.1%**  **-8.0%** | | |
| **Transplant** | | total  autogenic  allogenic  xenogenic  alloplastic | | 62,043 (24.1)  18,564 (7.2)  141 (0.05)  4,744 (1.8)  38,594 (15.0) | | 3,064 (23.6)  1,017 (7.8)  9 (0.07)  214 (1.6)  1,824 (14.1) | | 53,389 (23.1)  12,845 (5.6)  72 (0.03)  14,815 (6.4)  25,657 (11.1) | | 3,518 (23.4)  981 (6.5)  8 (0.05)  888 (5.9)  1,641 (10.9) | | -8,654 (-0.9)  -5,719 (-1.6)  -69 (-0.02)  +10,071 (+4.6)  -12,937 (-3.9) | | +454 (-0.2)  -36 (-1.3)  -1 (-0.01)  +674 (+4.3)  -183 (-3.1) | | **-3.9%**  **-22.7%**  **-42.9%**  **+248.8%**  **-25.8%** | | **-0.9%**  **-16.8%**  **-23.3%**  **+258.1%**  **-22.4%** | | |
| **Supplemental Table 3**  Two-step interventions with regard to their anatomical location and dichotomized by comorbidity burden defined by *van Walraven score* | | | | | | | | | | | | | | | | | | | |  |
|  |  | | **2009 - 2011** | | | | **2016 - 2018** | | | | **Absolute change** | | | | **Relative change** | | | | |  |
|  |  | | **vWs < 20** | | **vWS ≥ 20** | | **vWs < 20** | | **vWS ≥ 20** | | **vWs < 20** | | **vWS ≥ 20** | | **vWs < 20** | | **vWS ≥ 20** | |  |  |
| **All two-step interventions** |  | | **68,733** | | **3,989** | | **125,593** | | **10,082** | | **+56,860** | | **+6,093** | | **+82.7%** | | **+152.7%** | |  |  |
| **All endovascular interventions** |  | | **27,393** | | **1,614** | | **49,733** | | **3,958** | | **+21,794** | | **+2,344** | | **+78.0%** | | **+145.2%** | |  |  |
| **Angioplasty** | total  aorto-iliac  upper leg  below the knee  bypass graft | | 15,958 (23.2)  5,931 (8.6)  6,648 (9.7)  3,216 (4.7)  163 (0.2) | | 1,050 (26.3)  303 (7.6)  438 (11.0)  298 (7.5)  11 (0.3) | | 26,515 (21.1)  8,996 (7.2)  10,888 (8.7)  5,962 (4.7)  669 (0.5) | | 2,275 (22.6)  545 (5.4)  945 (9.4)  736 (7.3)  49 (0.5) | | +10,557 (-2.1)  +3,065 (-1.5)  +4,240 (-1.0)  +2,746 (+0.07)  +506 (+0.3) | | +1,225 (-3.8)  +242 (-2.2)  +507 (-1.6)  +438 (-0.2)  +38 (+0.2) | | **-9.0%**  **-17.0%**  **-10.4%**  **+1.5%**  **+124.6%** | | **-14.3%**  **-28.8%**  **-14.6%**  **-2.3%**  **+76.3%** | |  |  |
| **Drug-coated balloons** |  | | 127 (0.2) | | 6 (0.2) | | 3,872 (3.1) | | 260 (2.6) | | +3,745 (+2.9) | | +254 (+2.4) | | **+1568.5%** | | **+1614.5%** | |  |  |
| **Bare metal stent** | total  aorto-iliac  upper leg  below the knee  bypass graft | | 7,559 (11.0)  5,154 (7.5)  2,045 (3.0)  305 (0.4)  55 (0.08) | | 372 (9.3)  251 (6.3)  100 (2.5)  21 (0.5)  0 (0.0) | | 12,856 (10.2)  8,513 (6.8)  3,768 (3.0)  412 (0.3)  163 (0.1) | | 850 (8.4)  506 (5.0)  300 (3.0)  37 (0.4)  7 (0.1) | | +5,297 (-0.8)  +3,359 (-0.7)  +1,723 (+0.02)  +107 (-0.1)  +108 (+0.05) | | +478 (-0.9)  +255 (-1.3)  +200 (+0.5)  +16 (-0.2)  +7 (+0.1) | | **-6.9%**  **-9.6%**  **+0.8%**  **-26.1%**  **+62.2%** | | **-9.6%**  **-20.2%**  **+18.7%**  **-30.3%**  **n/a** | |  |  |
| **Drug-eluting stent** | total  aorto-iliac  upper leg  below the knee  bypass graft | | 134 (0.2)  61 (0.09)  48 (0.07)  25 (0.04)  0 (0.0) | | 0 (0.0)  0 (0.0)  0 (0.0)  0 (0.0)  0 (0.0) | | 611 (0.5)  261 (0.2)  196 (0.2)  154 (0.1)  0 (0.0) | | 44 (0.4)  16 (0.2)  9 (0.1)  19 (0.2)  0 (0.0) | | +477 (+0.3)  +200 (+0.1)  +148 (+0.1)  +129 (+0.1)  0 (0.0) | | +44 (+0.4)  +16 (+0.2)  +9 (+0.1)  +19 (+0.2)  0 (0.0) | | **+149.5%**  **+134.2%**  **+123.5%**  **+237.1%**  **n/a** | | **n/a**  **n/a**  **n/a**  **n/a**  **n/a** | |  |  |
| **Stent graft** | total  aorto-iliac  upper leg  below the knee  bypass graft | | 181 (0.3)  89 (0.1)  80 (0.1)  7 (0.01)  5 (0.01) | | 5 (0.1)  5 (0.1)  0 (0.0)  0 (0.0)  0 (0.0) | | 1,226 (1.0)  712 (0.6)  434 (0.3)  51 (0.04)  29 (0.02) | | 83 (0.8)  60 (0.6)  23 (0.2)  0 (0.0)  0 (0.0) | | +1,045 (+0.7)  +623 (+0.4)  +354 (+0.2)  +44 (+0.03)  +24 (+0.02) | | +78 (+0.7)  +55 (0.5)  +23 (+0.2)  0 (0.0)  0 (0.0) | | **+270.7%**  **+337.8%**  **+196.9%**  **+298.7%**  **+217.4%** | | **+556.8%**  **+374.8%**  **n/a**  **n/a**  **n/a** | |  |  |
| **Atherectomy** | total  aorto-iliac  upper leg  below the knee  bypass graft | | 67 (0.1)  15 (0.02)  38 (0.06)  14 (0.02)  0 (0.0) | | 0 (0.0)  0 (0.0)  0 (0.0)  0 (0.0)  0 (0.0) | | 59 (0.05)  20 (0.02)  39 (0.03)  0 (0.0)  0 (0.0) | | 0 (0.0)  0 (0.0)  0 (0.0)  0 (0.0)  0 (0.0) | | -8 (-0.05)  +5 (-0.005)  +1 (-0.02)  -14 (-0.02)  0 (0.0) | | 0 (0.0)  0 (0.0)  0 (0.0)  0 (0.0)  0 (0.0) | | **-51.8%**  **-27.0%**  **-43.8%**  **n/a**  **n/a** | | **n/a**  **n/a**  **n/a**  **n/a**  **n/a** | |  |  |
| **Thrombectomy (endovascular)** | total  aorto-iliac  upper leg  below the knee  bypass graft | | 489 (0.7)  33 (0.05)  206 (0.3)  219 (0.3)  31 (0.05) | | 23 (0.6)  0 (0.0)  9 (0.2)  14 (0.49)  0 (0.0) | | 834 (0.7)  54 (0.04)  345 (0.3)  389 (0.3)  46 (0.03) | | 92 (0.9)  0 (0.0)  44 (0.4)  42 (0.4)  6 (0.1) | | +345 (-0.04)  +21 (-0.005)  +139 (-0.03)  +170 (-0.009)  +15 (-0.008) | | +69 (+0.3)  0 (0.0)  +35 (+0.2)  +28 (+0.1)  +6 (+0.1) | | **-6.7%**  **-10.4%**  **-8.3%**  **-2.8%**  **-18.8%** | | **+58.3%**  **n/a**  **+93.4%**  **+18.7%**  **n/a** | |  |  |
| **Laser angioplasty** | total  aorto-iliac  upper leg  below the knee  bypass graft | | 97 (0.1)  11 (0.02)  65 (0.09)  21 (0.03)  0 (0.0) | | 0 (0.0)  0 (0.0)  0 (0.0)  0 (0.0)  0 (0.0) | | 140 (0.1)  22 (0.02)  80 (0.06)  38 (0.03)  0 (0.0) | | 5 (0.05)  0 (0.0)  0 (0.0)  5 (0.05)  0 (0.0) | | +43 (-0.03)  +11 (0.002)  +15 (-0.03)  +17 (-0.0003)  0 (0.0) | | 5 (0.05)  0 (0.0)  0 (0.0)  5 (0.05)  0 (0.0) | | **-21.0%**  **+9.5%**  **-32.6%**  **-1.0%**  **n/a** | | **n/a**  **n/a**  **n/a**  **n/a**  **n/a** | |  |  |
| **Thrombolysis** | total  aorto-iliac  upper leg  below the knee  bypass graft | | 2,644 (3.8)  92 (0.1)  1,218 (1.8)  1,092 (1.6)  242 (0.4) | | 158 (4.0)  7 (0.2)  77 (1.9)  65 (1.6)  9 (0.2) | | 2,520 (2.0)  67 (0.05)  1,040 (0.8)  1,233 (1.0)  180 (0.1) | | 247 (2.5)  6 (0.06)  94 (0.9)  122 (1.2)  25 (0.2) | | -124 (-1.8)  -25 (-0.08)  -178 (-0.9)  +141 (-0.6)  -62 (-0.2) | | +89 (-1.5)  -1 (-0.1)  +17 (-1.0)  +57 (-0.4)  +16 (+0.02) | | **-47.8%**  **-60.1%**  **-53.3%**  **-38.2%**  **-59.3%** | | **-38.1%**  **-66.1%**  **-51.7%**  **-25.7%**  **+9.9%** | |  |  |
| **Rotational thrombectomy** | total  aorto-iliac  upper leg  below the knee  bypass graft | | 98 (0.1)  10 (0.01)  62 (0.09)  18 (0.03)  8 (0.01) | | 0 (0.0)  0 (0.0)  0 (0.0)  0 (0.0)  0 (0.0) | | 988 (0.8)  88 (0.07)  622 (0.5)  247 (0.2)  31 (0.02) | | 82 (0.8)  6 (0.1)  43 (0.4)  28 (0.3)  5 (0.1) | | +890 (+0.6)  +78 (+0.06)  +560 (+0.4)  +229 (+0.2)  +23 (0.01) | | +82 (0.8)  +6 (0.06)  +43 (0.4)  +28 (0.3)  +5 (0.1) | | **+451.7%**  **+381.6%**  **+449.0%**  **+651.0%**  **+112.1%** | | **n/a**  **n/a**  **n/a**  **n/a**  **n/a** | |  |  |
| **Reentry Device** |  | | 39 (0.06) | | 0 (0.0) | | 112 (0.09) | | 20 (0.2) | | +73 (+0.03) | | 20 (0.2) | | **+57.2%** | | **n/a** | |  |  |
| **All surgical interventions** |  | | **41,340** | | **2,375** | | **75,860** | | **6,124** | | **+34,520** | | **+3,749** | | **+83.5%** | | **+157.9%** | |  |  |
| **Endarterectomy** | total  iliac  femoral  deep femoral  popliteal  lower leg  bypass graft | | 12,979 (18.9)  2,414 (3.5)  6,819 (9.9)  2,711 (3.9)  602 (0.9)  395 (0.6)  38 (0.06) | | 630 (15.8)  99 (2.5)  314 (7.9)  146 (3.7)  36 (0.9)  0 (0.0)  35 (0.9) | | 29,069 (23.1)  5,916 (4.7)  14,659 (11.7)  6,613 (5.3)  967 (0.7)  765 (0.6)  149 (0.1) | | 2,052 (20.4)  359 (3.6)  988 (9.8)  488 (4.8)  85 (0.8)  120 (1.2)  12 (0.1) | | +16,090 (+4.3)  +3,502 (+1.2)  +7,840 (+1.8)  +3,902 (+1.3)  +365 (-0.1)  +370 (+0.03)  +111 (+0.06) | | +1,422 (+4.6)  +260 (+1.1)  +674 (+1.9)  +342 (-+1.2)  +49 (-0.06)  +120 (+1.1)  -23 (-0.8) | | **+22.3%**  **+34.1%**  **+17.6%**  **+33.5%**  **-12.1%**  **+6.0%**  **+114.6%** | | **+28.9%**  **+43.5%**  **+24.5%**  **+32.2%**  **-6.6%**  **n/a**  **-86.4%** | |  |  |
| **Patch plastic** | total  femoral  deep femoral  popliteal  lower leg  bypass graft | | 4,344 (6.3)  2,660 (3.9)  1,064 (1.6)  271 (0.4)  326 (0.5)  23 (0.03) | | 205 (5.1)  111 (2.8)  71 (1.8)  10 (0.3)  0 (0.0)  13 (0.3) | | 8,257 (6.6)  5,343 (4.3)  1,669 (1.3)  505 (0.4)  548 (0.4)  192 (0.2) | | 629 (6.2)  375 (3.7)  116 (1.2)  46 (0.5)  74 (0.7)  18 (0.2) | | +3,913 (+0.3)  +2,683 (0.4)  +605 (-0.2)  +234 (+0.008)  +222 (-0.04)  +169 (+0.1) | | +424 (+1.1)  +264 (+0.9)  +45 (-0.6)  +36 (+0.2)  +74 (+0.7)  +5 (-0.1) | | **+4.1%**  **+9.9%**  **-14.2%**  **+2.0%**  **-8.0%**  **+356.8%** | | **+21.4%**  **+33.7%**  **-35.4%**  **+82.0%**  **n/a**  **-45.2%** | |  |  |
| **Embolectomy / Thrombectomy**  **(surgical)** | total  iliac  femoral  deep femoral  popliteal  lower leg  bypass graft | | 8,067 (11.7)  1,896 (2.8)  1,992 (2.9)  445 (0.6)  1,121 (1.6)  1,921 (2.8)  692 (1.0) | | 489 (12.3)  93 (2.3)  125 (3.1)  44 (1.1)  73 (1.8)  114 (2.9)  40 (1.0) | | 15,304 (12.2)  3,494 (2.8)  3,752 (3.0)  896 (0.7)  2,007 (1.6)  3,624 (2.9)  1,531 (1.2) | | 1,291 (12.8)  220 (2.2)  339 (3.4)  118 (1.2)  173 (1.7)  328 (3.3)  113 (1.1) | | +7,237 (+0.4)  +1,598 (+0.02)  +1,760 (+0.1)  +451 (+0.07)  +886 (-0.03)  +1,703 (+0.09)  +839 (+0.2) | | +802 (+0.5)  +127 (-0.1)  +214 (+0.2)  +74 (+0.07)  +100 (-0.1)  +214 (+0.4)  +73 (+0.1) | | **+3.8%**  **+0.9%**  **+3.1%**  **+10.2%**  **-2.0%**  **+3.2%**  **+21.1%** | | **+4.5%**  **-6.4%**  **+7.3%**  **+6.1%**  **-6.2%**  **+13.8%**  **+11.8%** | |  |  |
| **Bypass** | total  iliac origin  femoral origin  popliteal origin  upper leg target  lower leg target | | 7,617 (11.1)  730 (1.1)  6,157 (8.9)  730 (1.1)  3,659 (5.3)  3,958 (5.8) | | 566 (14.2)  36 (0.9)  463 (11.6)  67 (1.7)  238 (6.0)  328 (8.2) | | 8,832 (7.0)  862 (0.7)  7,092 (5.6)  878 (0.7)  4,162 (3.3)  4,670 (3.7) | | 978 (9.7)  53 (0.5)  813 (8.1)  112 (1.1)  371 (3.7)  607 (6.0) | | +1,215 (-4.1)  +132 (-0.4)  +935 (-3.3)  +148 (-0.4)  +503 (-2.0)  +712 (-2.0) | | +412 (-4.5)  +17 (-0.4)  +350 (-3.5)  +45 (-0.6)  +133 (-2.3)  +279 (-2.2) | | **-36.5%**  **-35.4%**  **-36.9%**  **-34.2%**  **-37.7%**  **-35.4%** | | **-31.6%**  **-41.8%**  **-30.5%**  **-33.9%**  **-38.3%**  **-26.8%** | |  |  |
| **Transplant** | total  autogenic  allogenic  xenogenic  alloplastic | | 8,333 (12.1)  2,570 (3.7)  16 (0.02)  800 (1.2)  4,947 (7.2) | | 485 (12.2)  171 (4.3)  0 (0.0)  48 (1.2)  266 (6.7) | | 14,398 (11.5)  2,807 (2.2)  33 (0.03)  6,039 (4.8)  5,519 (4.4) | | 1,174 (11.6)  286 (2.8)  0 (0.0)  429 (4.3)  459 (4.6) | | +6,065 (-0.7)  +237 (-1.5)  +17 (+0.003)  +5,239 (+3.6)  +572 (-2.8) | | +689 (-0.5)  +115 (-1.5)  0 (0.0)  +381 (+3.1)  +193 (-2.1) | | **-5.4%**  **-40.2%**  **+12.9%**  **+313.1%**  **-38.9%** | | **-4.2%**  **-33.8%**  **n/a**  **+253.6%**  **-31.7%** | |  |  |

| **Supplemental Table 4**  Hybrid interventions with regard to their anatomical location and dichotomized by comorbidity burden defined by *van Walraven score* | | | | | | | | | | |
| --- | --- | --- | --- | --- | --- | --- | --- | --- | --- | --- |
|  |  | **2009 - 2011** | | **2016 - 2018** | | **Absolute change** | | **Relative change** | | |
|  |  | **vWs < 20** | **vWS ≥ 20** | **vWs < 20** | **vWS ≥ 20** | **vWs < 20** | **vWS ≥ 20** | **vWs < 20** | **vWS ≥ 20** |  |
| **All hybrid interventions** |  | **37,560** | **1,651** | **84,619** | **5,400** | **+47,059** | **+3,749** | **+125.3%** | **+227.1%** |  |
| **All endovascular interventions** |  | **3,693** | **154** | **21,810** | **1,499** | **+18,117** | **+1,345** | **+490.6%** | **+873.4%** |  |
| **Angioplasty** | total  aorto-iliac  upper leg  below the knee  bypass graft | 2,204 (5.9)  849 (2.3)  905 (2.4)  428 (1.1)  22 (0.06) | 101 (6.1)  26 (1.6)  49 (3.0)  26 (1.6)  0 (0.0) | 11,584 (13.7)  4,316 (5.1)  4,717 (5.6)  2,200 (2.6)  351 (0.4) | 898 (16.6)  258 (4.8)  368 (6.8)  245 (4.5)  27 (0.5) | +9,380 (+7.8)  +3,467 (+2.8)  +3,812 (+3.2)  +1,772 (+1.5)  +329 (+0.4) | +797 (+10.5)  +232 (+3.2)  +319 (+3.8)  +219 (+3.0)  +27 (+0.5) | **+133.3%**  **+125.6%**  **+131.4%**  **+128.2%**  **+608.2%** | **+171.8%**  **+203.4%**  **+129.6%**  **+188.1%**  **n/a** |  |
| **Drug-coated balloons** |  | 62 (0.2) | 0 (0.0) | 2,211 (2.6) | 123 (2.3) | +2,149 (+2.4) | +123 (+2.3) | **+1482.9%** | **n/a** |  |
| **Bare metal stent** | total  aorto-iliac  upper leg  below the knee  bypass graft | 1,023 (2.7)  663 (1.8)  311 (0.8)  39 (0.1)  10 (0.03) | 44 (2.7)  27 (1.6)  17 (1.0)  0 (0.0)  0 (0.0) | 5,972 (7.1)  4,111 (4.9)  1,652 (1.9)  129 (0.2)  80 (0.09) | 355 (6.6)  233 (4.3)  106 (2.0)  16 (0.3)  0 (0.0) | +4,949 (+4.3)  +3,448 (+3.1)  +1,341 (+1.1)  +90 (+0.05)  +70 (+0.06) | +311 (+3.9)  +206 (+2.7)  +89 (+0.09)  +16 (+0.3)  0 (0.0) | **+159.1%**  **+175.2%**  **+135.8%**  **+46.8%**  **+255.1%** | **+146.7%**  **+163.8%**  **+90.6%**  **n/a**  **n/a** |  |
| **Drug-eluting stent** | total  aorto-iliac  upper leg  below the knee  bypass graft | 24 (0.06)  8 (0.02)  16 (0.04)  0 (0.0)  0 (0.0) | 0 (0.0)  0 (0.0)  0 (0.0)  0 (0.0)  0 (0.0) | 264 (0.3)  87 (0.1)  145 (0.2)  32 (0.04)  0 (0.0) | 5 (0.09)  5 (0.0)  0 (0.0)  0 (0.0)  0 (0.0) | +240 (+0.2)  +79 (+0.08)  +129 (+0.1)  +32 (+0.03)  0 (0.0) | +5 (+0.09)  +5 (+0.09)  0 (0.0)  0 (0.0)  0 (0.0) | **+388.3%**  **+382.7%**  **+302.3%**  **n/a**  **n/a** | **n/a**  **n/a**  **n/a**  **n/a**  **n/a** |  |
| **Stent graft** | total  aorto-iliac  upper leg  below the knee  bypass graft | 71 (0.2)  29 (0.08)  42 (0.1)  0  0 | 0 (0.0)  0 (0.0)  0 (0.0)  0 (0.0)  0 (0.0) | 653 (0.8)  358 (0.4)  267 (0.3)  17 (0.02)  11 (0.01) | 38 (0.7)  30 (0.6)  8 (0.1)  0 (0.0)  0 (0.0) | +582 (0.6)  +329 (0.3)  +225 (0.2)  +17 (0.02)  +11 (0.01) | +38 (+0.7)  +30 (+0.6)  +8 (+0.1)  0 (0.0)  0 (0.0) | **+308.2%**  **+448.0%**  **+182.2%**  **n/a**  **n/a** | **n/a**  **n/a**  **n/a**  **n/a**  **n/a** |  |
| **Atherectomy** | total  aorto-iliac  upper leg  below the knee  bypass graft | 6 (0.02)  0 (0.0)  6 (0.02)  0 (0.0)  0 (0.0) | 0 (0.0)  0 (0.0)  0 (0.0)  0 (0.0)  0 (0.0) | 44 (0.05)  10 (0.01)  34 (0.04)  0 (0.0)  0 (0.0) | 5 (0.09)  0 (0.0)  0 (0.0)  5 (0.09)  0 (0.0) | +38 (+0.04)  +10 (+0.01)  +28 (+0.02)  0 (0.0)  0 (0.0) | +5 (+0.09)  0 (0.0)  0 (0.0)  +5 (+0.09)  0 (0.0) | **+225.5%**  **n/a**  **+151.5%**  **n/a**  **n/a** | **n/a**  **n/a**  **n/a**  **n/a**  **n/a** |  |
| **Thrombectomy (endovascular)** | total  aorto-iliac  upper leg  below the knee  bypass graft | 31 (0.08)  0 (0.0)  12 (0.03)  19 (0.05)  0 (0.0) | 0 (0.0)  0 (0.0)  0 (0.0)  0 (0.0)  0 (0.0) | 156 (0.2)  20 (0.02)  49 (0.06)  69 (0.08)  18 (0.02) | 15 (0.3)  0 (0.0)  5 (0.09)  10 (0.2)  0 (0.0) | +125 (+0.1)  +20 (+0.02)  +37 (+0.03)  +50 (+0.03)  +18 (+0.02) | +15 (+0.3)  0 (0.0)  +5 (+0.09)  +10 (+0.2)  0 (0.0) | **+123.4%**  **n/a**  **+81.2%**  **+61.2%**  **n/a** | **n/a**  **n/a**  **n/a**  **n/a**  **n/a** |  |
| **Laser angioplasty** | total  aorto-iliac  upper leg  below the knee  bypass graft | 6 (0.02)  0 (0.0)  6 (0.02)  0 (0.0)  0 (0.0) | 0 (0.0)  0 (0.0)  0 (0.0)  0 (0.0)  0 (0.0) | 117 (0.1)  7 (0.01)  82 (0.09)  28 (0.03)  0 (0.0) | 0 (0.0)  0 (0.0)  0 (0.0)  0 (0.0)  0 (0.0) | +111 (+0.1)  +7 (+0.008)  +76 (+0.08)  +28 (+0.03)  0 (0.0) | 0 (0.0)  0 (0.0)  0 (0.0)  0 (0.0)  0 (0.0) | **+765.6%**  **n/a**  **+506.6%**  **n/a**  **n/a** | **n/a**  **n/a**  **n/a**  **n/a**  **n/a** |  |
| **Thrombolysis** | total  aorto-iliac  upper leg  below the knee  bypass graft | 248 (0.7)  12 (0.03)  95 (0.3)  122 (0.3)  19 (0.05) | 9 (0.5)  0 (0.0)  0 (0.0)  9 (0.5)  0 (0.0) | 546 (0.6)  17 (0.02)  170 (0.2)  317 (0.4)  42 (0.05) | 51 (0.9)  0 (0.0)  23 (0.4)  28 (0.5)  0 (0.0) | +298 (-0.02)  +5 (-0.01)  +75 (-0.05)  +195 (+0.05)  +23 (-0.001) | +42 (+0.4)  0 (0.0)  +23 (+0.4)  +19 (-0.03)  0 (0.0) | **-2.3%**  **-37.1%**  **-20.6%**  **+15.3%**  **-1.9%** | **+73.3%**  **n/a**  **n/a**  **-4.9%**  **n/a** |  |
| **Rotational thrombectomy** | total  aorto-iliac  upper leg  below the knee  bypass graft | 10 (0.03)  0 (0.0)  10 (0.03)  0 (0.0)  0 (0.0) | 0 (0.0)  0 (0.0)  0 (0.0)  0 (0.0)  0 (0.0) | 225 (0.3)  24 (0.03)  149 (0.2)  28 (0.03)  24 (0.03) | 0 (0.0)  0 (0.0)  0 (0.0)  0 (0.0)  0 (0.0) | +215 (+0.2)  +24 (+0.03)  +139 (+0.1)  +28 (+0.03)  +24 (+0.03) | 0 (0.0)  0 (0.0)  0 (0.0)  0 (0.0)  0 (0.0) | **+898.7%**  **n/a**  **+561.4%**  **n/a**  **n/a** | **n/a**  **n/a**  **n/a**  **n/a**  **n/a** |  |
| **Reentry Device** |  | 8 (0.02) | 0 (0.0) | 38 (0.04) | 9 (0.2) | +30 (+0.02) | +9 (0.2) | **+110.8%** | **n/a** |  |
| **All surgical interventions** |  | **33,867** | **1,497** | **62,809** | **3,901** | **+28,942** | **+2,404** | **+85.5%** | **+160.6%** |  |
| **Endarterectomy** | total  iliac  femoral  deep femoral  popliteal  lower leg  bypass graft | 13,809 (36.8)  3,072 (8.2)  7,153 (19.0)  3,039 (8.1)  339 (0.9)  159 (0.4)  47 (0.1) | 516 (31.3)  120 (7.3)  256 (15.5)  118 (7.1)  17 (1.0)  5 (0.3)  0 (0.0) | 27,723 (32.8)  6,447 (7.6)  13,575 (16.0)  6,562 (7.8)  575 (0.7)  362 (0.4)  157 (0.2) | 1,521 (28.2)  306 (5.7)  753 (13.9)  369 (6.8)  53 (1.0)  27 (0.5)  13 (0.2) | +13,914 (-4.0)  +3,375 (-0.6)  +6,422 (-3.0)  +3,523 (-0.3)  +236 (-0.2)  +203 (+0.004)  +110 (+0.06) | +1,005 (3.1)  +186 (-1.6)  +497 (-1.6)  +251 (-0.3)  +36 (-0.05)  +22 (+0.2)  +13 (+0.2) | **-10.9%**  **-6.8%**  **-15.8%**  **-4.2%**  **-24.7%**  **+1.1%**  **+48.3%** | **-9.9%**  **-22.0%**  **-10.1%**  **-4.4%**  **-4.7%**  **+65.1%**  **n/a** |  |
| **Patch plastic** | total  femoral  deep femoral  popliteal  lower leg  bypass graft | 3,836 (10.2)  2,725 (7.3)  868 (2.3)  128 (0.3)  88 (0.2)  27 (0.07) | 125 (7.6)  90 (5.5)  35 (2.1)  0 (0.0)  0 (0.0)  0 (0.0) | 6,741 (7.9)  4,737 (5.6)  1,348 (1.6)  236 (0.3)  255 (0.3)  165 (0.2) | 419 (7.8)  289 (5.4)  100 (1.9)  11 (0.2)  13 (0.2)  6 (0.1) | +2,905 (-2.2)  +2,012 (-1.7)  +480 (-0.7)  +108 (-0.06)  +167 (+0.06)  +138 (+0.1) | +294 (+0.2)  +199 (-0.1)  +65 (-0.3)  +11 (+0.2)  +13 (+0.2)  +6 (+0.1) | **-22.0%**  **-22.8%**  **-31.1%**  **-18.2%**  **+28.6%**  **+171.3%** | **+2.5%**  **-1.8%**  **-12.6%**  **n/a**  **n/a**  **n/a** |  |
| **Embolectomy / Thrombectomy**  **(surgical)** | total  iliac  femoral  deep femoral  popliteal  lower leg  bypass graft | 7,049 (18.8)  1,818 (4.8)  2,264 (6.0)  319 (0.8)  1,137 (3.0)  959 (2.6)  552 (1.5) | 341 (20.7)  90 (5.5)  102 (6.2)  19 (1.2)  51 (3.1)  51 (3.1)  28 (1.7) | 11,467 (13.6)  3,229 (3.8)  3,184 (3.8)  694 (0.8)  1,441 (1.7)  1,684 (2.0)  1,235 (1.5) | 782 (14.5)  190 (3.5)  218 (4.0)  51 (0.9)  108 (2.0)  129 (2.4)  86 (1.6) | +4,418 (-5.2)  +1,411 (-1.0)  +920 (-2.2)  +375 (-0.03)  +304 (-1.3)  +725 (-0.6)  +683 (-0.01) | +441 (-6.2)  +100 (-1.9)  +116 (-2.1)  +32 (-0.2)  +57 (-0.1)  +78 (-0.7)  +58 (-0.1) | **-27.8%**  **-21.2%**  **-37.6%**  **-3.4%**  **-43.7%**  **-22.1%**  **-0.7%** | **-29.9%**  **-35.5%**  **-34.7%**  **-17.9%**  **-35.3%**  **-22.7%**  **-6.1%** |  |
| **Bypass** | total  iliac origin  femoral origin  popliteal origin  upper leg target  lower leg target | 3,125 (8.3)  420 (1.1)  2,585 (6.9)  120 (0.3)  2,134 (5.7)  991 (2.6) | 210 (12.7)  23 (1.4)  180 (10.9)  7 (0.4)  127 (7.7)  83 (5.0) | 4,564 (5.4)  669 (0.8)  3,725 (4.4)  170 (0.2)  3,023 (3.6)  1,532 (1.8) | 406 (7.5)  60 (1.1)  329 (6.1)  17 (0.3)  254 (4.7)  152 (2.8) | +1,439 (-2.9)  +249 (-0.3)  +1,140 (-2.5)  +50 (-0.1)  +889 (-2.1)  +541 (-0.8) | +196 (-5.2)  +37 (-0.3)  +149 (-4.8)  +10 (-0.1)  +127 (-3.0)  +69 (-2.2) | **-35.2%**  **-29.3%**  **-36.0%**  **-37.1%**  **-37.1%**  **-31.4%** | **-40.9%**  **-20.2%**  **-44.1%**  **-25.7%**  **-38.9%**  **-44.0%** |  |
| **Transplant** | total  autogenic  allogenic  xenogenic  alloplastic | 6,048 (16.1)  1,268 (3.4)  36 (0.09)  1,049 (2.8)  3,695 (9.8) | 305 (18.5)  93 (5.6)  0 (0.0)  38 (2.3)  174 (10.5) | 12,314 (14.6)  1,536 (1.8)  31 (0.03)  6,266 (7.4)  4,481 (5.3) | 773 (14.3)  111 (2.1)  0 (0.0)  362 (6.7)  300 (5.6) | +6,266 (-1.5)  +268 (-1.5)  -5 (-0.06)  +5,217 (+4.6)  +786 (-4.5) | +468 (-4.2)  +18 (-3.6)  0 (0.0)  +324 (+4.4)  +126 (-5.0) | **-9.6%**  **-46.2%**  **-61.8%**  **+165.1%**  **-46.2%** | **-22.5%**  **-63.5%**  **n/a**  **+191.3%**  **-47.3%** |  |
